# Evidence of cyclical light/dark-regulated expression of freezing tolerance in young winter wheat plants

**DOI:** 10.1371/journal.pone.0198042

**Published:** 2018-06-18

**Authors:** Daniel Z. Skinner, Brian Bellinger, William Hiscox, Gregory L. Helms

**Affiliations:** 1 Department of Crop and Soil Sciences, Washington State University, Pullman, Washington, United States of America, US Department of Agriculture, Agricultural Research Service, Wheat Health, Genetics and Quality Research Unit, Pullman, Washington, United States of America; 2 The Center for NMR Spectroscopy, Washington State University, Pullman, Washington, United States of America; NARO Institute of Agrobiologial Sciences, JAPAN

## Abstract

The ability of winter wheat (*Triticum aestivum* L.) plants to develop freezing tolerance through cold acclimation is a complex rait that responds to many environmental cues including day length and temperature. A large part of the freezing tolerance is conditioned by the C-repeat binding factor (CBF) gene regulon. We investigated whether the level of freezing tolerance of 12 winter wheat lines varied throughout the day and night in plants grown under a constant low temperature and a 12-hour photoperiod. Freezing tolerance was significantly greater (P<0.0001) when exposure to subfreezing temperatures began at the midpoint of the light period, or the midpoint of the dark period, compared to the end of either period, with an average of 21.3% improvement in survival. Thus, freezing survival was related to the photoperiod, but cycled from low, to high, to low within each 12-hour light period and within each 12-hour dark period, indicating ultradian cyclic variation of freezing tolerance. Quantitative real-time PCR analysis of expression levels of CBF genes 14 and 15 indicated that expression of these two genes also varied cyclically, but essentially 180° out of phase with each other. Proton nuclear magnetic resonance analysis (^1^H-NMR) showed that the chemical composition of the wheat plants' cellular fluid varied diurnally, with consistent separation of the light and dark phases of growth. A compound identified as glutamine was consistently found in greater concentration in a strongly freezing-tolerant wheat line, compared to moderately and poorly freezing-tolerant lines. The glutamine also varied in ultradian fashion in the freezing-tolerant wheat line, consistent with the ultradian variation in freezing tolerance, but did not vary in the less-tolerant lines. These results suggest at least two distinct signaling pathways, one conditioning freezing tolerance in the light, and one conditioning freezing tolerance in the dark; both are at least partially under the control of the CBF regulon.

## Introduction

Many plant functions appear to cycle from low to high levels of activity on approximately a 24-hour schedule, a phenomenon recognized centuries ago [1]. Virtually every physiological process in a plant undergoes at least one rhythmic cycle within a 24-hour period [2]. Many of these diurnal cycles appear to be a response to external time cues, such as the light/dark or high/low temperature cycles that naturally arise from the alternation of day and night. These environmental time cues, termed Zeitgebers (German for “time givers”), tend to entrain the internal timing systems of plants to a period of about 24 h, corresponding to the period of the earth’s rotation [1,3,4]. While some processes tend to reach peak activity in the middle of the subjective day or night, others reach peak activity at characteristic time points other than the middle [5]. The alternating phases of the cycles are accompanied by extensive reengineering of the transcriptome [3], indicating a great many genes are primarily or secondarily influenced by the clock mechanisms.

Cycles with a period of less than 24 h, or ultradian cycles, are becoming increasingly well-known in plants and are thought to have a number of functions including regulation of cell signaling, optimization of energy efficiency, optimization of responses to stimuli, spatial organization, temporal organization, the separation of incompatible processes occurring in the same subcellular compartment, and coordination of processes occurring in separate compartments [6]. One school of thought holds that diurnal cycles arose through consolidation of ultradian cycles [6], presumably because of an evolutionary advantage associated with longer cycle times. Cycles with periods as short as a few minutes occur in plants as nutrients are taken up in the roots [2], and as part of carbon assimilation and partitioning during photosynthesis-related metabolism [7].

Specifically, in relation to freezing tolerance in wheat (*Triticum aestivum* L.) plants, expression levels of many of the C-repeat binding factor (CBF) genes were observed to fluctuate in diurnal fashion in plants grown under a 16 hours light/8 hours dark photoperiod at 20°C [8]. The CBF genes encode transcription factors that are intricately involved in the conditioning of the response to low temperature, as well as other stress factors [9]. In Arabidopsis, CBF genes expression has been shown to cycle in diurnal fashion [10–12]. Most of the 13 wheat CBF genes examined by Badawi et al. [8] reached minimum expression at the end of an 8-hour dark period, then reached maximum expression 10–12 hours after the start of the light period. One of the CBF genes (TaCBFIIIa-D6) also showed a second, cyclic increase in expression at about the middle of the 8-hour dark period [8]. At least 65 CBF genes are present in the wheat genome [13] and involvement of these genes in both above- and below-freezing low temperature tolerance of wheat has been amply demonstrated [14–17]. Campoli et al. [14] observed that variation in expression levels of some of wheat, rye (*Secale cereale* L.), and barley (*Hordeum vulgare* L.) CBF genes were reduced during the dark phase of various photoperiod regimes, leading the authors to suggest their expression “may reflect a temperature-dependent, light-regulated diurnal response.” That observation, and the observations of Badawi et al. [8] of diurnal cycles of expression of several wheat CBF genes suggests that low temperature tolerance may also show cyclic variation in expression. Others have reported that the photoperiod significantly affects the freezing tolerance of some wheat lines [18,19] and references therein).

The wheat genes CBF14 and CBF15 have become of particular interest to freezing tolerance investigations. These genes are part of a cluster of at least 11 CBF genes, collectively referred to as the *Fr*2 locus, found on the long arm of the group 5 homoeologous chromosomes of hexaploid wheat [17, 20–22]. While all of these CBF genes presumably contribute to freezing tolerance, CBF14 and CBF15 are expressed to the highest levels of all of the genes in the *Fr*2 locus on exposure to low temperature [21], perhaps indicating they condition a greater portion of the observed freezing tolerance. Copy number variation of CBF14 [22] and CBF15 [23] has been shown to be a significant factor influencing the effectiveness of these genes with greater numbers of copies associated with greater freezing tolerance. Each of these lines of evidence suggest that CBF14 and CBF15 play a major role in freezing tolerance. Indeed, Soltesz et al. [24] demonstrated that genetically transforming these genes individually into freezing-sensitive spring barley plants resulted in highly significant improvement of freezing tolerance. Novak et al., [25] found that light quality also strongly influences CBF14 expression and subsequently reported [26] that “it can be assumed that temperature and light signals are relayed to the level of CBF14 expression via separate signalling routes”.

Therefore, it appears that varying light conditions may influence the expression of the CBF genes, especially CBF 14 and CBF 15, even at constant temperature, implying that freezing tolerance also may vary over the course of 24 hours at constant temperature. Accordingly, the objective of this study was to determine whether fully cold-acclimated winter wheat plants grown under diurnal light/dark cycles were significantly more freezing tolerant at the mid-points, or at the end-points of the light and dark periods, and to assess the expression levels of CBF14 and CBF15 at these time points to look for a possible relationship of expression of these genes and freezing tolerance.

## Materials and methods

### Freezing tolerance of wheat plants

The winter wheat cultivars included in this study were ‘Eltan’, ‘Froid’, ‘Norstar’, ‘Bruehl’, ‘Centurk 78’, ‘Jagger’, ‘Lewjain’, ‘Madsen’, ‘Masami’, ‘Nugaines’, and ‘Tiber’, and germplasm line ‘Oregon Feed Wheat #5’ (ORFW). These cultivars represent a range of freezing tolerance with Norstar the most freezing tolerant and ORFW the least. The freezing tolerance of most of these lines were described previously [27,28]. The ORFW germplasm line appears to carry a major, dominant gene conditioning freezing sensitivity [29].

Seeds were sown into Sunshine Mix LC1 planting medium (Sun Gro Horticulture, Bellevue, WA, USA) in 6-container packs (Model 1020, Blackmore Co., Belleville, MI, USA); the capacity of each container in the pack was about 100 ml. Each freezing tolerance trial was comprised of eight of the 6-container packs. Each of the 12 wheat lines were represented four times in each freezing trial. Each freezing trial was considered a replication and the four representations of each line were considered subsamples within trials. Seeds were germinated and seedlings grown at 22°C in a growth chamber (Model E15, Conviron, Pembina, ND) under cool, white fluorescent lights (about 300 µmol m^-2^ s^-1^ PAR at the soil surface) with a 12-hour photoperiod until the seedlings reached the three-leaf stage. Relative humidity was not controlled. The plants were then transferred to 4°C with a 12-hour photoperiod (about 250 µmol m^-2^ s^-1^ PAR at mid-plant height) for 5 weeks to induce cold acclimation prior to freezing survival tests. Lights were turned on at 6 a.m. and turned off at 6 p.m. Dawn is often defined as “ZT0” (Zeitgeber time zero; [1]), thus, in this study, ZT0 refers to 6a.m. Plants were irrigated weekly with nutrient solution containing macro and micronutrients (Peters Professional, Scotts Co., Camarillo, CA, USA). Prior to freezing, the flats were drenched with a solution of 10mg/L Snomax snow inducer (Snomax LLC, International, Centennial, CO, USA) maintained at 4°C, and allowed to drain until drainage had essentially ceased, then freezing was carried out in darkness in a programmable freezer (model LU-113, Espec Corp., Hudsonville, MI, USA). The temperature of the plant growth medium in each container near the crowns of the plants was monitored using thermistor-based, food piercing temperature probes with expected accuracy of ±0.3°C, and an internet-enabled temperature monitor (Model E-16, Sensatronics, Bow, NH, USA). The temperature was recorded every 2 min using a data capture script running on a remote computer. Freezing tests were initiated at ZT0, ZT6, ZT12 and ZT18, i.e. at the start, midpoint, and end of the 12-hour light and dark periods. The trials initiated at ZT18 (middle of the dark period) were placed in the freezer at ZT12, at the start of the dark period, and the freezer was maintained at 4°C until the start of the freezing test at ZT18, thereby avoiding exposure to light before the start of the freezing trial. At the start of each freezing test, the temperature in the freezer was reduced from 4°C to -3°C and held at -3°C for 16 hours to allow all of the soil water to be converted to ice and the heat of ice formation to completely dissipate from the samples before the temperature was lowered to the target temperature at a rate of -2°C h^-1^. The temperature was held at the target for 2 hours, then raised to 4°C at a rate of 2°C h^-1^. Plants were exposed in separate trials to target temperatures of -13.5, -14.5, -15.5, or -16.5°C with freezing tests to each target temperature started at each of the four time points, ZT0, ZT6, ZT12 and ZT18. A total of 1,723 containers holding 37,236 plants were tested. After freezing, the plants were then transferred to a growth chamber at 4°C for 24 hours to allow the soil to completely thaw and the soil temperature to equilibrate, then were moved to 22°C with a 16-hour photoperiod for recovery. Survival was scored after 5 weeks of regrowth. Statistical analysis was carried out using the “Fit Model” platform of JMP version 12 (SAS Institute Inc., Cary, NC, USA). The proportions of plants surviving formed the response variable and wheat lines (genotypes), minimum temperature, freezing trial start time, replications, and subsamples within replications were the predictor variables. Means separation was determined using least significant difference with a significance level of 0.05. For statistical analysis of the main effects, the response was expressed as the arcsine of the square root of the surviving proportion as recommended [30] but are expressed in the original scale in this report. For calculations of the LT_50_, the temperature expected to result in death of 50% of the plants, the response was expressed as the ratio of the number of plants surviving to the number of plants frozen in each trial, estimated with binomial distribution and probit link function specified. The minimum temperatures for the LT_50_ calculations were specified as the minimum temperature recorded for each container, rounded to the nearest 0.5°C, then increased by 30°C to avoid negative numbers in the probit calculations. The LT_50_ values are expressed in the original scale in this report.

### CBF gene expression analysis

At each of the time points, ZT0, ZT6, ZT12 and ZT18, seedlings of the wheat lines Norstar, Tiber and ORFW that had been grown to 5 weeks of cold acclimation at constant 4° C under a 12-hour photoperiod as described above were quickly removed from the soil and plunged into liquid nitrogen. Three independent biological samples grown at different times were collected. Total RNA was extracted from the crown tissue (meristematic regions) using Trizol reagent and the supplier-recommended procedure (ThermoFisher Scientific, Waltham, MA, USA), and then treated with DNase I and further purified using the Direct-zol RNA MiniPrep kit (Zymo Research, Irvine, CA, USA). Purified RNA was quantified using a Nanodrop 1000C instrument (ThermoFisher Scientific, Waltham, MA, USA). Complementary DNA (cDNA) was synthesized using the ProtoScript First Strand cDNA Synthesis Kit (New England Biolabs, Ipswich, MA, USA) using 1µg RNA as starting template. The resulting cDNA was diluted 1:5 prior to use as template for quantitative real-time PCR. The qPCRs were conducted on an Applied Biosystems 7300 Real-Time PCR System (ThermoFisher Scientific, Waltham, MA, USA) using 25µl preparations that consisted of 1X Go-Tag Colorless Master Mix (Promega, Madison, WI, USA), 1µl cDNA, 200µM each primer, 0.85X SYBR Green I as a reporter fluor, and 300nM ROX dye (ThermoFisher Scientific, Waltham, MA, USA) as a passive, internal fluorescence standard. The PCR primers used for CBF14 were: forward, 5'-AACCGATGACGAGAAGGAAA-3', and reverse, 5'-AACTCCGAGTAGCACGATCC-3' [24]. The primers used for CBF15 were: forward, 5’-GTCGTCCATGGAAAATACCG-3’, and reverse: 5’-ATGTGTCCAGGTCCATTTC-3’ [23].

The PCR amplification profile was 3 min at 95°C, then 45 cycles of 95°C for 30 sec, 55°C for 25 sec, and 72°C for 30 sec. Two technical replications of each of the three biological replications were performed. The qPCR software available with the instrument was used to determine the Ct, the fractional cycle number at which the fluorescence intensity reached an arbitrary threshold, using the threshold determined by the software. Relative fold change was determined using the delta-delta Ct method [31], with Ct values normalized to the Ct values of house-keeping gene Ta30797, encoding phosphogluconate dehydrogenase, using the forward primer 5’-GCCGTGTCCATGCCAGTG-3’, and reverse 5’-TTAGCCTGAACCACCTGTGC-3’, as described by elsewhere [32].

### NMR spectra of plant extracts

Wheat plants of the cultivars Norstar, ORFW and Tiber were grown and cold-acclimated for 5 wks at 4°C and 12-hour photoperiod as described above. After 5 wks, the plants (about 20 plants) were removed from the cell packs at each of the ZT0, 6, 12, or 18 time points, quickly rinsed in ice water, then dropped into liquid nitrogen. Each combination of wheat cultivar and time of day was represented by two biological replications grown and processed at separate times. Roots, residual caryopses, and shoots above the first node were removed while the plant tissue was frozen, using forceps, and the crown and “stem” tissue was ground to a fine powder with a mortar and pestle and additional liquid nitrogen. The powdered tissue was transferred to a 15 ml screw-top tube and transferred to a water bath at 85°C before the tissue had thawed. Samples were maintained at 85°C for 15 min to stop intrinsic enzyme activity, then held on ice until all samples were processed. An 18 gauge (1.3mm diameter) hypodermic needle was used to punch a hole in the bottom of the 15 ml tube, which was then placed within a 50 ml centrifuge tube. The samples were centrifuged at 8,000 xG for 10 minutes and the exudate recovered from the 50 ml tube. The residual plant tissue was transferred to a new 15 ml screw-top tube, 2 ml of pure water were added, samples were mixed by vortexing, then placed in a boiling water bath for 15 min. Samples were then cooled to room temperature, centrifuged at 10,0000 x G for 10 min, and the supernatant was transferred to a new tube. The recovered plant exudate and supernatant samples were frozen to -20°C and then lyophilized to complete dryness. The dried plant material was dissolved in deuterium oxide (D_2_O; Cambridge Isotope Laboratories, Tewksbury, MA, USA) to a concentration of 50 mg/ml. In preparation for nuclear magnetic resonance (NMR) analysis, samples were diluted to 15 mg/ml in 1mM TMSP (2,2,3,3-d4 sodium-3-trimethylsilylpropionate; Cambridge Isotope Laboratories) in D_2_O. ^1^H NMR spectra were recorded on a Varian / Agilent 400-MR (399.763 MHz for ^`^H) instrument using a Varian OneNMR probe. The data were acquired using the standard PRESAT pulse sequence where the carrier was set to the residual water frequency. A relaxation delay of 13 seconds was followed by a weak presaturation field (30 Hz B1) for two seconds and the data collected using a 90-degree pulse and an acquisition time of 2.6 seconds. Sixteen NMR scans were completed for each sample. The receiver gain was kept constant for all samples.

All spectra were processed using the DataChord Spectrum Miner software (One Moon Scientific, Westfield NJ). The raw FIDs were loaded into DataChord Spectrum Miner and processed as a batch with no apodization and the resulting spectra were aligned using the peak from TMSP as the standard located at 0 ppm. Spectral regions were defined manually and integrated peak areas determined (S1 Table). The compound peak areas of all spectra were standardized to TMSP as follows. The grand mean area of all of the TMSP peaks in the experiment were determined, then for each run, the area of the TMSP peak was divided by that grand mean, yielding a standardizing factor for the run. Each peak area in that spectrum was multiplied by the factor to yield standardized data. Further analysis was carried out using JMP software (http://www.jmp.com).

Identification of metabolites was undertaken by analysis of multidimensional NMR data and comparison of chemical shift data with database values. Two-dimensional ^1^H-^1^H TOCSY (TOtal Correlation SpectroscopY), ^1^H-^13^C HSQC (Heteronuclear Single Quantum Correlation) and ^1^H-^13^C HMBC (Heteronuclear Multiple Bond Correlation) spectra were recorded using a sample that contained a compound associated with freezing tolerance based on PCA analysis. Two-dimensional data were recorded on a Varian / Agilent VNMRS 500 (^1^H 499.84 MHz, ^13^C 125.69 MHz) using the standard pulse programs zTOCSY, gHSQCAD and gHMBCAD. For the TOCSY data 2 x 512 t1 increments (States-TPPI) using 16 scans each were acquired using non-uniform data sampling (NUS) at 50% density giving a total acquisition time of 4.25 hours. Residual water was reduced using a 20 Hz presaturation radio frequency field for 1 second and the TOCSY mixing was accomplished using a DIPSI-2 sequence for 80 ms. The sweep width was 5,660 Hz in both dimensions and the data were processed using DataChord Spectrum Analyst (One Moon Scientific, Westfield, NJ) using an iterative soft threshold Fourier Transform algorithm to produce a final 2Kx2K point 2-dimensional spectrum. HSQC data were acquired using gradient coherence selection and utilizing 2 x 256 t1 increments of 64 scans and NUS sampling (50%) for a total acquisition time of 7.75 hours. Residual water was reduced with presaturation and the sweep width in the ^1^H dimension was 5660 Hz while for the ^13^C dimension 20,736 Hz (165 ppm) was used. Data were processed similarly to the TOCSY data to yield a 2K x 2K final spectrum. HMBC data were recorded with 2 x 256 t1 increments using linear sampling and 64 scans per t1 increment to give a total acquisition time of 13.3 hours. A spectral with of 5,660 Hz was used for the ^1^H dimension and 24,510 Hz (195 ppm) was used for the ^13^C dimension. A long-range ^1^H-^13^C J-coupling of 8 Hz was used to develop ^1^H-^13^C correlations. Residual water was suppressed with presaturation and the data processed to produce a 2K x 2K final spectrum. The data were anodized in the t2 domain with a sine bell function and in the t1 domain with a gaussian function and the data were represented in the mixed phase mode (absolute value in F2 and phase sensitive mode in F1). Once identifications were obtained samples were compared with authentic samples of compounds.

## Results

### Plant freezing tolerance

**Fig 1:**
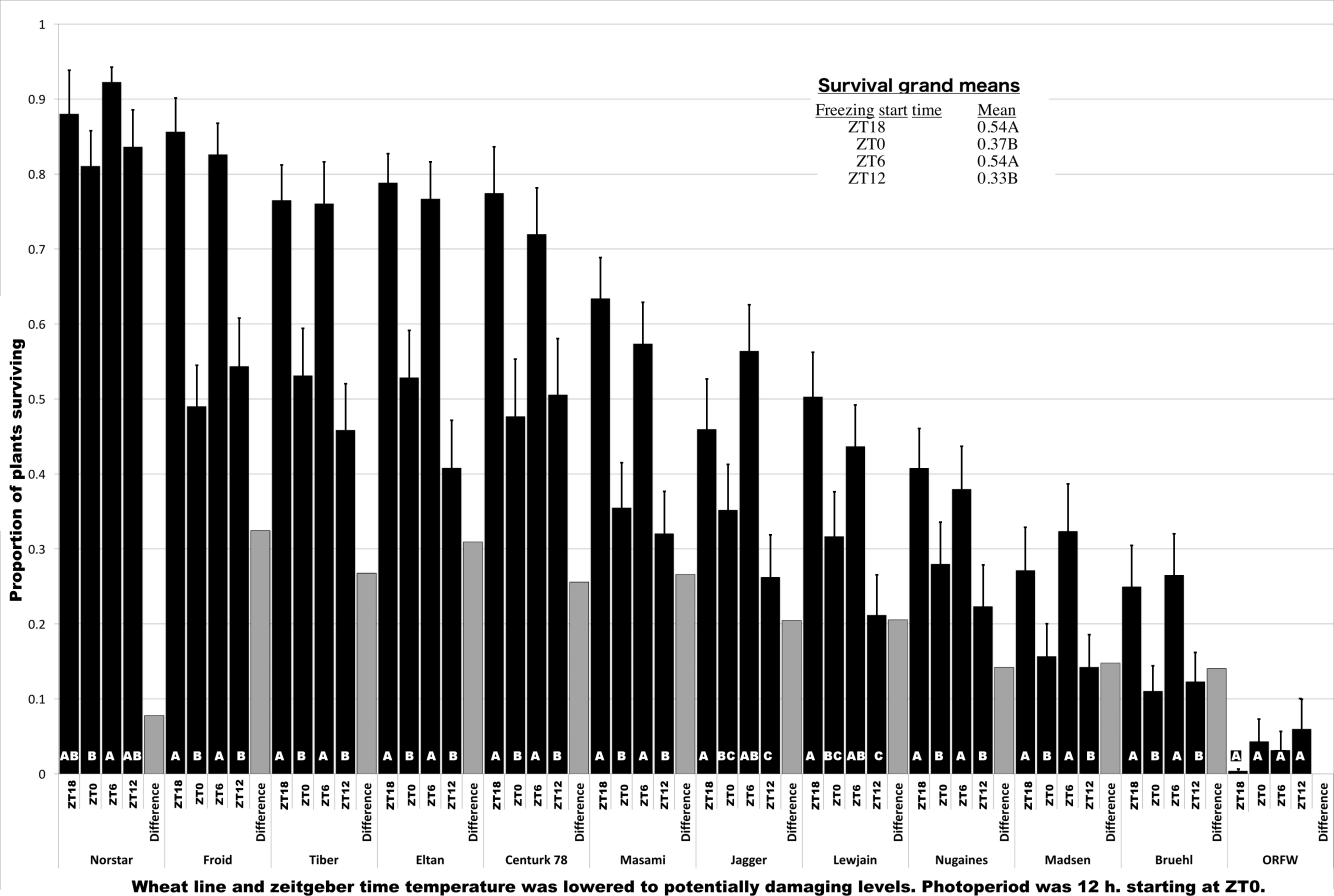
Survival of winter wheat plants acclimated to the cold at 4°C while growing under a 12 h light/12 h dark photoperiod for 5 weeks and then exposed to subzero temperatures. The proportion of plant survival was obtained by the average of all survival values of wheat plants treated to target temperatures of -13.5, -14.5, -15.5 and -16.5°C. Exposure to the subzero temperatures was initiated at ZT0 (zietgeber time 0, the start of the light period), ZT6, ZT12 (the start of the dark period), or ZT18. Bars with the same white letter at the bottom indicate survival proportions that were not significantly different within the indicated wheat line (P = 0.05). The grey bars labeled “Difference” indicate the difference between the averages of the two mid-period trials compared to the start-of-period trials within the indicated wheat line. The inset shows the mean survival proportions of all wheat lines and statistical separation. Error bars indicate one standard error unit. Wheat lines are sorted in decreasing order of mean survival.

Tabulated survival data are provided in S2 Table. Analysis of variance revealed that wheat lines (genotypes), minimum temperature, and the time the freezing tests were initiated all were significant sources of variation at P<0.0001 (Table 1). Replications and subsamples within replications were significant only at P<0.05, and together accounted for less than 1.5% of the variation (Table 1). Average survival of each of the wheat lines when freezing was initiated at each of the time points (ZT0, ZT6, ZT12, ZT18) is shown in Fig 1. These times corresponded to the start and midpoint of the light period (ZT0 and ZT6, respectively), and the start and midpoint of the dark period (ZT12 and ZT18, respectively). With the exception of ORFW, which had very little survival in any of the tests, freezing tolerance was significantly greater when the plants were exposed to the subzero temperatures at the midpoint of the light period or the midpoint of the dark period, compared to the start of either of those periods (Fig 1). The improvement of survival in the tests conducted mid-period ranged from 7.8 to 32.4%, compared to the tests initiated at the start of the periods, with an average improvement of 21.3% (shown by the grey bars in Fig 1). LT_50_ values calculated from the combined data for the trials that were started at the midpoint of the light and dark periods, and for the trials initiated at the start of those periods are shown in Table 2. The differences in LT_50_ between the periods ranged from 1.8 to 4.9°C, with an average of 2.5°C (Table 2).

**Table 1:** Analysis of variance of freezing survival.

Source of variation	Degrees of freedom	Sum of squares	F ratio	Probability of greater F	Proportion of variance explained
**Wheat Lines**	11	198.9	115.3	<0.0001	0.33
**Minimum temperature**	3	96.4	153.7	<0.0001	0.16
**Trial start time**	3	32.1	68.2	<0.0001	0.053
**Replications**	4	1.9	3.0	0.02	0.003
**Subsamples within Replications**	23	5.8	1.6	0.04	0.010
**Total**	1722	603.4			

Freezing tolerance measured was of winter wheat plants grown under a 12-hour photoperiod at constant 4°C for 5 weeks, and then exposed to a range of subfreezing temperatures. Freezing tolerance trials were initiated at the beginning and midpoints of the light and dark phases.

**Table 2:** Origin, market class, and LT_50_ of 12 winter wheat lines when freezing tolerance was assessed after 5 weeks of cold acclimation at the midpoint or the end of alternating 12 hour light and dark cycles.

			LT_50_ (°C) when assessed at this part of the light or dark phase		
Wheat line	Origin	Market Class^a^	Midpoint	End	LT_50_ Dif-ference (°C)
**Norstar**	Saskatchewan, Canada	HRWW	-18.9	-17.1	1.8
**Froid**	Montana, USA	HRWW	-16.1	-14.3	1.8
**Tiber**	Montana, USA	HRWW	-15.5	-12.5	3.0
**Eltan**	Washington, USA	SWWW	-15.8	-10.8	4.9
**Centurk 78**	Nebraska, USA	HRWW	-15.5	-13.6	1.9
**Masami**	Washington, USA	SWWW	-14.6	-12.8	1.8
**Jagger**	Kansas, USA	HRWW	-14.3	-12.2	2.1
**Lewjain**	Washington, USA	SWWW	-13.7	-11.5	2.2
**Nugaines**	Washington, USA	SWWW	-13.5	-9.4	4.1
**Madsen**	Washington, USA	SWWW	-13.6	-11.0	2.6
**Bruehl**	Washington, USA	SWWCW	-12.7	-10.9	1.8
**ORFW**	Oregon, USA	SWWW	-9.4	not estimable	

^a^HRWW hard red winter wheat, SWWW soft white winter wheat, SWWCW soft white winter club wheat.

All freezing tolerance tests were initiated with a 16-hour period at -3°C to allow the soil water to freeze and the heat of ice formation to dissipate, followed by controlled reduction in temperature to the target, potentially damaging, challenge temperature, as described above. This 16-hour “pre-freezing” period and the controlled reduction in temperature to the target resulted in the plants experiencing the potentially damaging temperature at about 24 hours after the start of the test. Consequently, the plants were exposed to the lowest temperature at about the same time of day as the test was started, but 24 hours later.

### CBF14 and CBF15 expression

Expression of CBF14 in Norstar and Tiber showed upregulation from ZT6 to the subsequent ZT0, then downregulation from ZT0 to ZT6 (Table 3). In contrast, expression of CBF14 in ORFW was upregulated from ZT12 until the subsequent ZT0, then downregulated during the ZT0 to ZT12 time interval (Table 3).

**Table 3:** Fold-change of expression^a^ of two CBF genes over 24 hours in three winter wheat lines grown at constant 4°C and 12 hours light/12 hours dark photoperiod.

		Fold change over time interval^b^			
Gene	Wheat Line	ZT0-ZT6	ZT6-ZT12	ZT12-ZT18	ZT18-ZT0
CBF14	Norstar	-8.79	1.38	2.45	2.60
CBF14	Tiber	-2.04	1.82	1.07	1.05
CBF14	ORFW	-1.32	-1.36	1.68	1.07
CBF15	Norstar	3.49	-3.85	17.50	-15.9
CBF15	Tiber	1.08	-2.40	6.18	-2.80
CBF15	ORFW	-7.32	-1.77	4.99	2.60

^a^Expression fold-change measurements were determined using the delta-delta Ct analysis method of quantitative real-time PCR data with a phosphogluconate dehydrogenase gene used as the constant expression standard.

^b^ZT0 (Zeitgeber Time 0) indicates subjective dawn, the start of the light phase. The dark phase began at ZT12.

Regulation of CBF15 in ORFW was very similar to regulation of CBF14 (Table 3). In contrast, in Norstar and Tiber, the regulation of CBF15 was essentially 180° out of sync with CBF14, *i*.*e*. CBF15 was downregulated from ZT6 to ZT12 and again from ZT18 to ZT0 while CBF14 was upregulated, and CBF15 was upregulated from ZT0 to ZT6 while CBF14 was downregulated during that time interval (Table 3).

### NMR spectra of plant extracts

**Fig 2:**
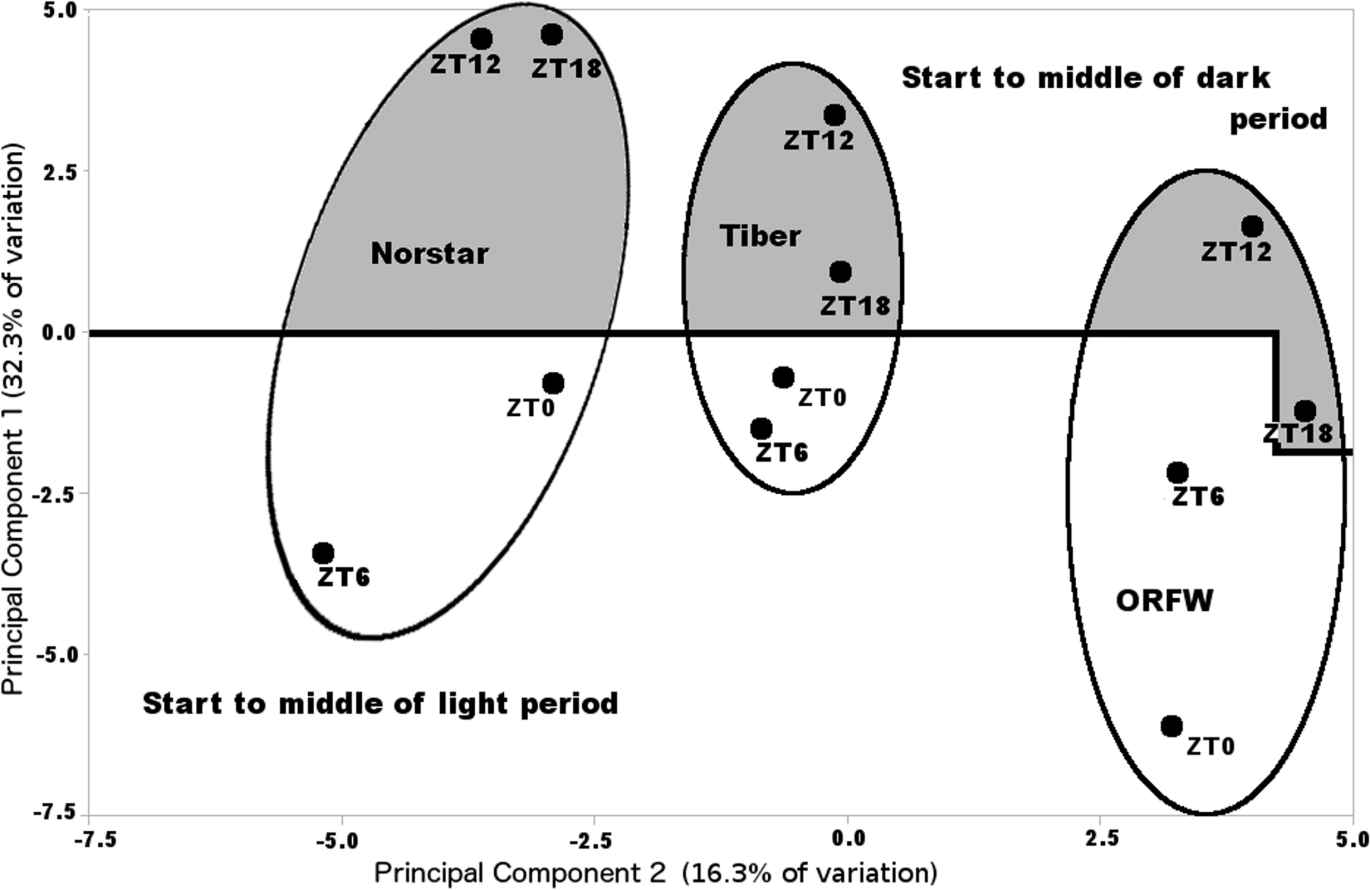
Principal component graph based on nuclear magnetic resonance (NMR) spectra of cellular fluid from wheat plants. Fluid was extracted from wheat lines Norstar, Tiber or Oregon Feed Wheat #5 (ORFW), at each of four time points ZT0 (Zeitgeber time 0, start of light period), ZT6, ZT12 (start of dark period) or ZT18. Principal components were based on standardized concentrations of 70 compounds identified in the NMR spectra from each wheat line at each time point.

An example of the ^1^H NMR spectra obtained is shown in S1 Fig. A total of 70 compounds ranging from 1 ppm (the TMSP standard) to 9.5 ppm were identified in all of the samples, as shown in S1 Table. For overall comparison of the plant lines and times of sample collection, the data from the two kinds of extractions (exudate vs. boiled) were considered as one data set and analyzed with principal component analysis (PCA). A plot of the first two principal components clearly distinguished the three plant lines (Fig 2), indicating Norstar, Tiber and ORFW differed from one another in relative content of some of the 70 compounds identified. The four sample collection time points also were associated with consistent positions on the PCA chart (Fig 2). The data points associated with the end of the light period/start of the dark period, ZT12, and middle of the dark period, ZT18, were clearly separated from the data points associated with the end of the dark period/start of the light period, ZT0, and the middle of the light period, ZT6 in each of the samples (Fig 2). These results suggested that the cellular contents of the plants were constantly in flux throughout the 24-hour cycle, at constant 4°C and 12-hour light/12-hour dark photoperiod. However, in contrast to the 12-hour cycling of freezing tolerance (Fig 1), cycling of the metabolites detected with NMR was in diurnal fashion with data points associated with the dark period (ZT12 to ZT18) consistently distinct from data points associated with the light period (ZT0 to ZT6) on the PCA plot (Fig 2). Thus, there did not appear to be an overall similarity of metabolite dynamics as detected by NMR (Fig 2), with the ultradian variation in freezing tolerance (Fig 1). This lack of association was most apparent when considering that the decreased levels of freezing tolerance were observed with freezing tests initiated at ZT0 or ZT12, which consistently were widely separated on the PCA plot of the NMR spectra (Fig 2).

**Fig 3:**
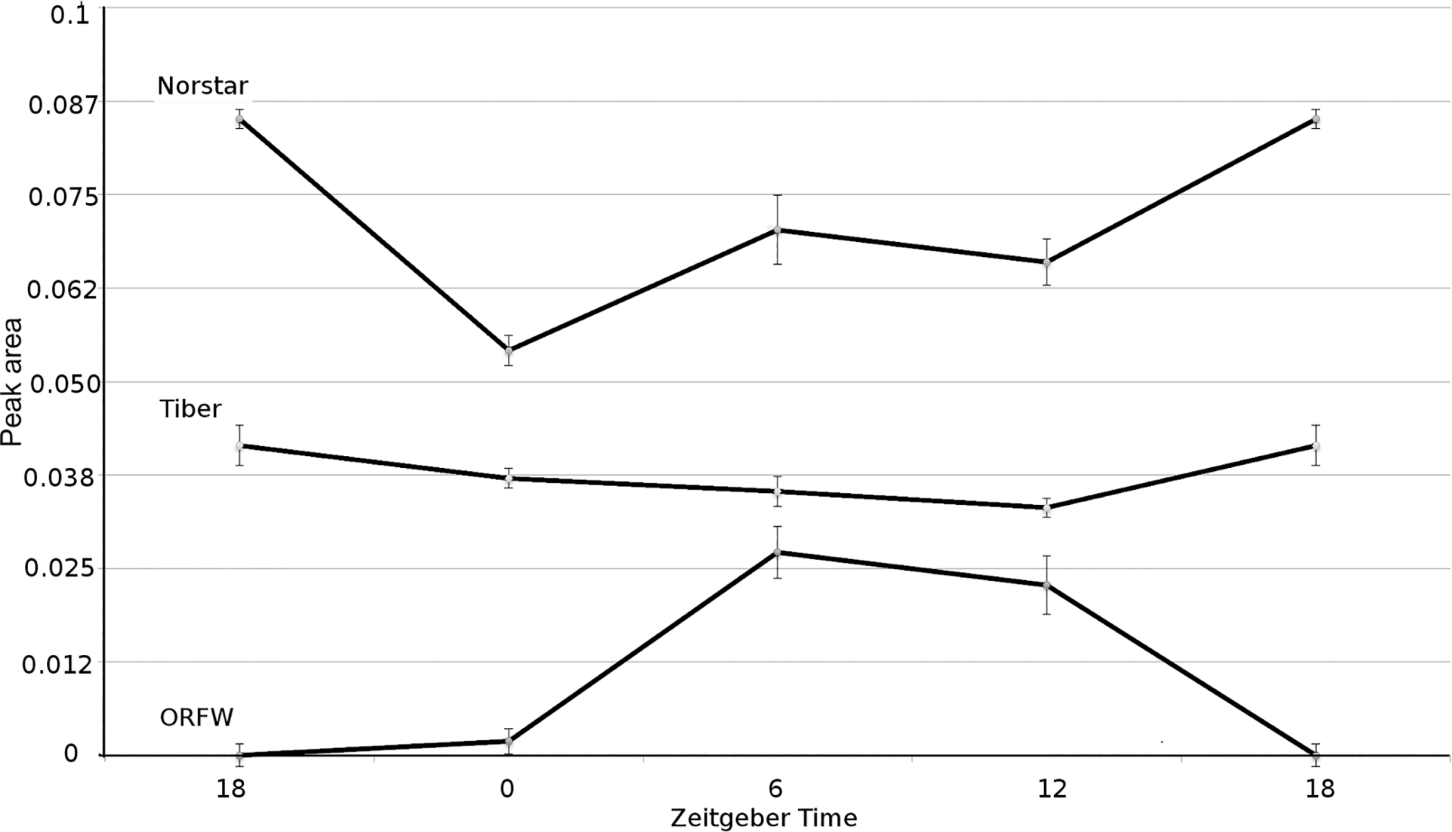
Concentration dynamics of a compound tentatively identified as glutamine in nuclear magnetic resonance (NMR) spectra of cellular fluid extracted from wheat plants. The wheat lines Norstar, Tiber and Oregon Feed Wheat #5 (ORFW) were grown at 4°C under a 12-hour photoperiod for 5 weeks. Samples for NMR analysis were extracted at each of four time points ZT0 (Zeitgeber time 0, start of light period), ZT6, ZT12 (start of dark period) or ZT18. The midpoint of the dark period (ZT18) is shown at both ends of the graph in order to show the changes in concentration from the start (ZT12) to the end (ZT0) of the dark period. Error bars indicate ± 1 standard error unit (n = 6).

The PCA plot represents overall similarity of the composition of the plant extracts and may not represent the behavior of individual compounds. Further examination of the concentration dynamics of the 70 individual compounds found in the NMR spectra from each plant line at each time point revealed that “compound 15”, observed as a peak at 2.06 to 2.19 ppm (S1 Table), varied in a manner that was consistent with expressed freezing tolerance (Fig 3). Of the three lines examined with NMR, Norstar was the most freezing tolerant, Tiber was intermediate, and ORFW was the least freezing tolerant at each of the time points (Fig 1). At each of the time points, the concentration of compound 15 was greatest in Norstar, intermediate in Tiber, and lowest in ORFW (Fig 3). Also, in Norstar, the concentration of compound 15 varied in ultradian fashion, reaching local maxima at ZT6 and ZT18 and local minima at ZT0 and ZT12 (Fig 3), coincident with levels of expressed freezing tolerance (Fig 1). In contrast, the concentration of compound 15 remained essentially unchanged in Tiber throughout the 24-hour period at 50–70% of the concentration seen in Norstar (Fig 3). In ORFW, with very little freezing tolerance (Fig 1), compound 15 reached maximum concentration at ZT6, at about 40% of the concentration seen in Norstar, but then declined to an undetectable level by ZT18, and increased only slightly between ZT18 and ZT0 (Fig 3). Thus, there appeared to be an association of the concentration of compound 15 with the observed relative levels of freezing tolerance in these three wheat lines, especially apparent with consistently higher concentrations and cyclic variation in parallel with variation of freezing tolerance in Norstar.

**Fig 4:**
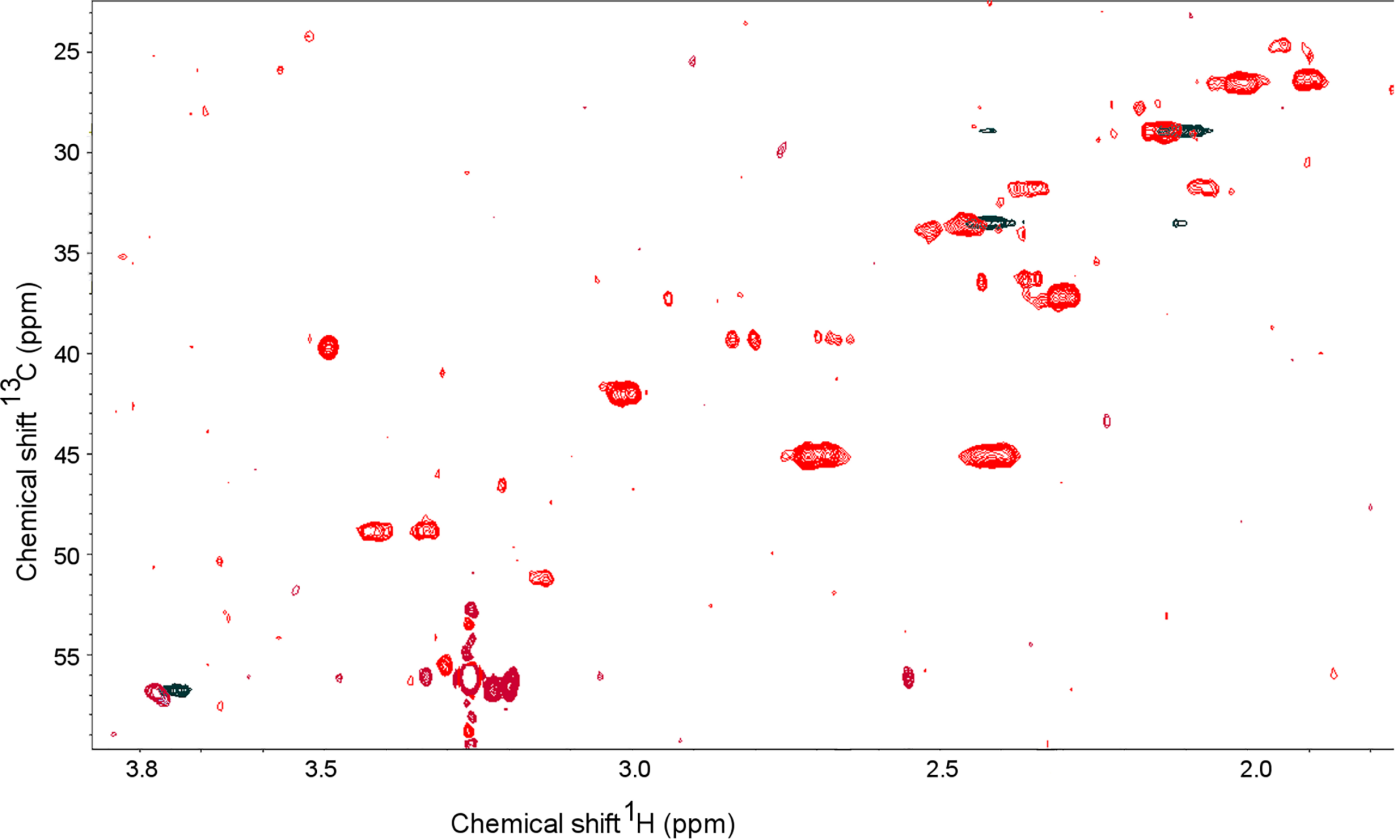
Heteronuclear single quantum correlation (HSQC) of ^1^H and 13C signals comparing components of wheat plant cellular fluid to glutamine. The plant fluid components are shown as red contours, glutamine is shown as black contours. The chemical shifts in 13C from glutamine are coincident with signals from the unknown wheat compound (compound 15) and shifts in the ^1^H dimension are just 2 Hz shifted from the authentic sample, probably due to minute pH or concentration differences.

The identity of compound 15 was revealed by analysis of several 2-dimensional data sets starting with the TOCSY data where the signal at 2.144 ppm in the proton spectrum correlated to a signal at 2.452 ppm and further to another at 3.777 ppm. The HSQC data showed the signal at 2.144 ppm to be a methylene group with a carbon chemical shift of 29.0 ppm whereas the signal at 2.452 ppm was another methylene with a carbon chemical shift of 33.7 ppm and the signal at 3.777 ppm was a methine with a carbon chemical shift of 56.8 ppm. The HMBC data showed that the methylene at 2.144 ppm had long-range correlation to two carbonyl groups at 176.7 and 180.3 ppm. The methine at 3.777 ppm was also correlated by a long-range coupling to the carbonyl at 176.7 ppm. Long-range correlations were seen between the methylene groups and the methine group but no correlations could be seen from the methylene at 2.452 ppm to the carbonyl groups. The signal to noise ratio of the HMBC data was not high due to the overall low concentration of most of the metabolites in the mixture so even with the lack of correlation from one of the methylene groups to one or more carbonyls it was concluded that the patterns matched closely with those expected for glutamic acid or glutamine. HSQC spectra were collected on both glutamic acid and glutamine in D_2_O and compared to the spectra recorded on the plant sample. The overlay of the data sets for the plant sample and for glutamine are shown in Fig 4. The chemical shifts in ^13^C from glutamine were coincident with compound 15, while signals in the ^1^H dimension from compound 15 were slightly shifted (2 Hz) from the authentic sample (Fig 4). This small of a difference easily could be due to minute pH or concentration differences, hence, we suggest that compound 15 is glutamine.

## Discussion

Freezing tolerance of young, fully cold-acclimated plants of the 12 winter wheat lines investigated here was expressed in a biphasic manner in plants grown at constant 4°C under a 12-hour photoperiod. Of the four time points studied, the greatest freezing tolerance occurred at ZT6, the midpoint of the light phase, and ZT18, the midpoint of the dark phase (Fig 1). This ultradian cycling of phenotypic expression of freezing tolerance suggested that genes involved in freezing tolerance also were expressed in ultradian cycles. The transcript expression levels of CBF14 and CBF15, genes shown to play a major role in freezing tolerance in wheat [17] were shown to cycle throughout the day, but essentially 180° out of phase with one another, in freezing tolerant cultivars Norstar and Tiber (Table 3). However, in wheat line ORFW, a line that develops very little freezing tolerance (Fig 1), the cyclic expression levels of CBF14 and CBF15 were essentially in phase (Table 3). Thus, although both CBF14 and CBF15 appeared to be upregulated from ZT12 to the subsequent ZT0 in ORFW (Table 3), there was no evidence of greater freezing tolerance as a result of that simultaneous elevated expression of both genes (Fig 1). In the case of Norstar and Tiber, freezing tolerance manifested most strongly at ZT6, when CBF15 was expressed to high levels, concomitant with low levels of expression of CBF14, and at ZT18, when both genes were expressed to high levels (Table 3). These results suggest that precise coordination of expression of these genes, as opposed to simply higher levels of expression, was critical to manifestation of greater freezing tolerance in these two wheat lines.

We previously found that ORFW is capable of responding to freezing stress in some ways similar to Norstar and Tiber. For example, lipid dynamics in response to six low-temperature treatments were very similar in ORFW and five other cultivars, including Norstar and Tiber [33], indicating that some components of the low-temperature response of the cold-sensitive ORFW function similarly to those components in more cold-tolerant genotypes. This observation suggests that different pathways may significantly contribute to freezing tolerance in the different genotypes. The evidence of the involvement of glutamine may point to one such pathway, suggesting that Norstar may make use of a glutamine-dependent pathway that is not functional in Tiber or ORFW.

Others [34,35] reported increased glutamine concentration in wheat plants on exposure to low temperatures. Kan et al, [36] reported that supplying glutamine to rice plants grown in hydroponic culture resulted in upregulation of several genes involved in stress response including a number of transcription factors, leading the authors to suggest that “glutamine may also function as a signaling molecule to regulate gene expression in plants.” Kamada-Nobusada et al. [37] also suggested glutamine may function as a signal molecule in rice plants, and Miranda et al. [38] and Molina-Rueda and Kirby [39] reported that glutamine may function as a signal molecule in stress response in *Sorghum* species and *Pinus* species, respectively. Thus, a growing body of evidence suggests that plants have the potential to activate a stress response pathway that may be regulated by variation in glutamine concentration.

In the three wheat lines studied here, the concentration of glutamine in the cellular fluid varied in concentration in a manner consistent with freezing tolerance in that the concentration was greatest in Norstar, the most freezing tolerant line, intermediate in Tiber, which is intermediate in freezing tolerance, and lowest (sometimes undetectable) in ORFW, which is the least cold tolerant line (Fig 3). Also, the concentration of glutamine in Norstar varied with two highs and two lows in the 24-hour period (Fig 3), coincident with the freezing tolerance dynamics of Norstar (Fig 1). Others have found diurnal fluctuation in the concentration of some amino acids in wheat sap under warm (20–22°C) conditions and a 16-hour photoperiod, but glutamine concentration was not found to vary in that study [40]. That result, considered with the results reported here, suggests that glutamine may function as a component of the pathway(s) responsible for cyclic variation of freezing tolerance under certain conditions of temperature and photoperiod in genotypes capable of using that pathway; constant low temperature (4°C) and 12-hour photoperiod in the cultivar Norstar in the present case.

The observed sequential increase, then decrease in freezing tolerance is consistent with our previous findings showing that exposing the plants to -3°C resulted in sequential, extensive reengineering of the transcriptome [16], and of cellular composition [33], accompanied by significant increases in freezing tolerance [41]. Others have shown that ice crystal formation begins at about -3°C in wheat tissues [42] suggesting that the variation in expression of freezing tolerance at the time points studied (Fig 1) probably originated in this initial response to -3°C and ice crystal formation. Plant biological rhythms entrained to day length usually persist for several cycles after the plants are moved to darkness [43] and thus the biphasic response we observed (Fig 1) resulted from interaction of gene products present at the time the plants were exposed to -3°C and darkness, interacting with the gene products and altered cellular component composition involved in the response that developed over the -3°C incubation period and subsequent exposure to lower temperature, likely while the two cycles observed within the 24-hour time frame continued to function. This biphasic stress response pattern is similar to that described for genes encoding enzymes involved in response to high light stress in Arabidopsis grown under a 16 hours light, 8 hours dark photoperiod [44]. Of the genes investigated in that study, two peaks of expression were found, one in the light and a second in the dark [44]. The authors suggested that the gene expression “was controlled by light and a second unidentified factor” [44].

Our observations of expression dynamics of CBF14 and CBF15 under low temperature (4° C) and 12-hour photoperiod were consistent with the possibility that cohorts of CBF genes, and consequently genes of the CBF regulon, were expressed in a ultradian fashion, but with the expression dynamics of some out of phase with others in some genotypes (Table 3), leading to the ultradian variation in freezing tolerance we observed (Fig 1). Under this hypothesis, freezing tolerance would appear to be under the control of “light and a second unidentified factor” as described in Arabidopsis [44], but the second factor simply may be the lack of light, to which some regulatory genes, including CBF genes [8], respond with upregulation. In barley, at least 20 CBF genes have been found, and have been classified into three phylogenetic groups, designated group 1, 3 or 4 [45]. Under a 12-hour photoperiod and constant 22°C, expression of CBF genes from group 1 was not detected [46]. Expression of CBF genes from group 4 cycled in diurnal fashion, reaching peak expression 8–12 hours after subjective dawn [46]. Expression of CBF genes from group 3 cycled in diurnal fashion, but one of them reached peak expression at the start of the dark period while expression of a second gene from group 3 reached peak expression near the end of the dark period [46]. These observations are consistent with the ultradian cycling of freezing tolerance we observed (Fig 1), again suggesting that the onset of the light phase, and the onset of the dark phase were characterized by specific regulation of unique cohorts of genes conditioning freezing tolerance, with the tolerance ultimately reaching its greatest expression at the midpoint of the light, and of the dark phases.

It is likely that this kind of regulation is dependent on the temperature at which the plants are grown in addition to the photoperiod, as has been shown with other wheat genes. For example, expression levels of wheat genes encoding lipocalins and similar proteins are correlated with the capacity to develop freezing tolerance; some of these genes in wheat plants grown under 16 hours light, 8 hours dark reached a peak of expression during the dark phase when grown at 4°C, but not when grown at 20°C [47].

Whether the pathways active in the increased freezing tolerance observed after exposure to sub-freezing temperature beginning at the midpoint of the dark phase were the same pathways acting after exposure to sub-freezing temperature beginning at the midpoint of the light phase is unknown. However, Bieniawska et al., [48] investigated the effects of low temperature and diurnal cycling on the Arabidopsis cold-responsive transcriptome and found “stronger, more abundant induction of TFs in the morning than in the evening,” (TFs = transcription factors); consistent with different transcription factors and presumably different target genes being involved in the response to cold at different time points of the light cycle. Thus, we suggest that the 12-hour cycle from low, to high, to low freezing tolerance in the light, and the similar cycle that completed in 12 hours of darkness, represent distinct, possibly overlapping signaling pathways that function as 12-hour ultradian cycles, and these pathways may well be components of the CBF regulon. In addition, specific genotypes may activate additional pathway(s), perhaps modulated by glutamine in some genotypes, that contribute to the realized freezing tolerance. This complexity of the freezing tolerance response, and apparently different pathways functioning in different genotypes, is not surprising considering the genetic constitution of the wheat plant. Wheat is an allohexaploid species formed by naturally-occurring interspecific hybridization of three diploid ancestors, first to form a tetraploid species from two of the diploids and then hybridization of the tetraploid with a third diploid to form the allohexaploid [49]. The hybridization event that led to the allohexaploid occurred relatively recently, perhaps less than 10,000 years ago [49]. Hence, each of the three genomes (designated A, B, or D [49]) was contributed by a species that itself was subject to stress-related selection pressures for many generations, including low temperature stress, and presumably developed tolerance mechanisms independently of the other species. The three genomes comprising *T*. *aestivum* still contain “many (distinct) functional gene complexes” [49] and interact in complex ways with myriad consequences [49]. These complex interactions include “gene silencing, gene elimination, or gene activation and transposon activation via genetic and epigenetic alterations” [49]. Transposon activation was observed during the cold acclimation process in Norstar, but not three other, closely-related, less cold-tolerant wheat lines [50]. The uniqueness of this response to Norstar, among four closely-related wheat lines, suggests the specific kinds of complex interactions that occur among the three genomes is unique to each individual allohexaploid genome. This possibility is consistent with our observations of differing regulation of CBF14 and CBF15 (Table 3) and glutamine concentration (Fig 3) among three wheat lines with differing freezing tolerance, perhaps indicating that the observed freezing tolerance was predominantly determined by a different genome (A, B, or D) in each line.

Further study of the effects of environmental factors on the expression of the freezing tolerance that results from these pathways is needed to reveal the genes and mechanisms involved. From a practical standpoint, these results suggest that tests of freezing tolerance and of gene expression dynamics related to freezing tolerance in winter wheat lines should be standardized to the photoperiod in order to meaningfully compare the relative levels of freezing tolerance among wheat lines.

## Supporting information

S1 Fig Example ^1^H NMR spectrum of wheat plant cellular fluid. (TIF)

S1 Table Standardized peak intensities of ^1^H-NMR signals from young wheat plant cellular fluid. (CSV)

S2 Table Freezing survival data of young wheat plants. (CSV)
