# Supplementary figures and images for: Evidence of cyclical light/dark-regulated expression of freezing tolerance in young winter wheat plants
Source: PLoS One. 2018 Jun 18;13(6):e0198042. doi: 10.1371/journal.pone.0198042 (PMC6005534; doi:10.1371/journal.pone.0198042)

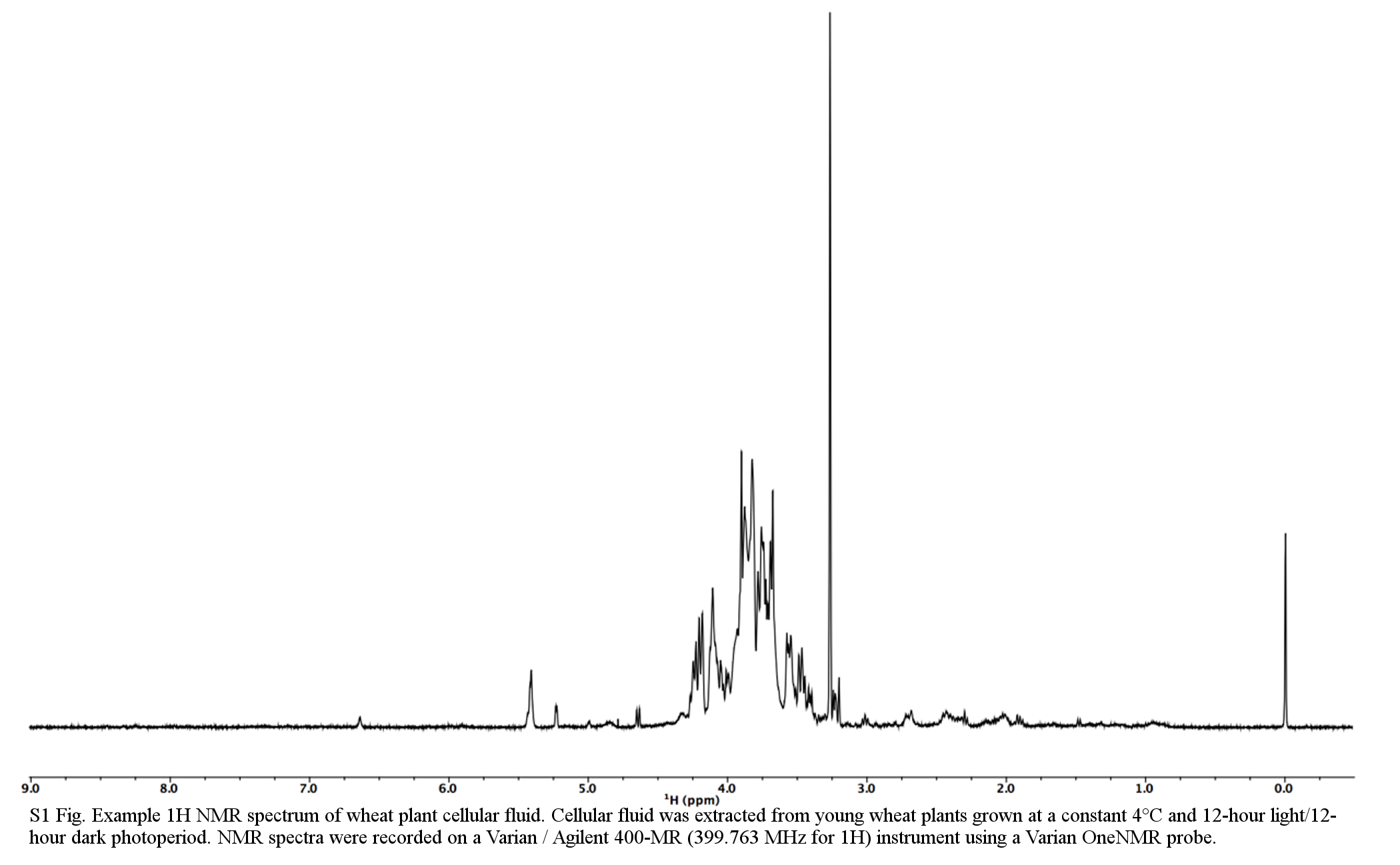

Supplement: S1 Fig — (TIF) [file pone.0198042.s001.tif]
